# Chronic Osteomyelitis of the Jaws: Management and Outcomes in a Tertiary Maxillofacial Surgery Unit

**DOI:** 10.3390/cmtr18040043

**Published:** 2025-10-15

**Authors:** Patrícia Santos, Carolina Moreira, Nuno Gião, Paulo Valejo Coelho

**Affiliations:** 1 Department of Head and Neck, Portuguese Institute of Oncology Francisco Gentil, 1099-023 Lisbon, Portugal; 2 São José Local Health Unit, Department of Maxillofacial Surgery, 1150-199 Lisbon, Portugal; 3 São José Local Health Unit, Department of Pathology, 1150-199 Lisbon, Portugal; 4 NOVA Medical School, Universidade Nova de Lisboa, 1169-056 Lisbon, Portugal

**Keywords:** osteomyelitis jaws, osteonecrosis, osteoradionecrosis, odontogenic bone disease

## Abstract

Objective: This study aims to evaluate the management and outcomes over 14 years at a tertiary maxillofacial surgery unit. Methods: Retrospective cohort study of patients from a Portuguese tertiary center of maxillofacial surgery with histopathologically confirmed diagnoses of chronic osteomyelitis of the jaws between January 2010 and December 2023. Demographic and clinical characteristics, treatment, and progression of the disease were evaluated. Results: Fifty-three patients were included—28 women (52.8%), mean age 55 (95% CI 5–90) years. The mandible was affected in 84.9% (*n* = 45) of cases. Secondary chronic osteomyelitis was diagnosed in 88.7% (*n* = 47), with medication-related osteonecrosis of the jaw (MRONJ) being the most common etiology (38.3%). Bacteriological samples were contributory in 52% (*n* = 13) and 46.1% (*n* = 6) were resistant to amoxicillin. All received antibiotics for a median time of 27.3 days. Surgical treatment included sequestrectomy (*n* = 40, 75.5%), marginal (*n* = 5, 9.4%), and segmental mandibulectomy (*n* = 8, 15.1%). Clinical remission was achieved in 77.4% (*n* = 41) of cases with higher success in MRONJ (*n* = 15, 83.3%) than ORN (*n* = 4, 57.1%). Conclusions: Almost half of the isolates were amoxicillin-resistant, reinforcing the need for susceptibility testing. Surgical management guided by etiology and disease stage remains essential, with more extensive resection needed in MRONJ and ORN.

## 1. Introduction

Osteomyelitis of the jaws is an inflammatory condition affecting the entire bone, including the marrow, and occurs more commonly in the mandible than the maxilla. Unlike osteomyelitis in long bones and other skeletal sites, jaw osteomyelitis has distinct characteristics due to the presence of teeth, unique oral microbiota, and varying vascularity [1,2].

Currently, this disease is predominantly a chronic disorder, associated with significant functional impairment in severe cases. The rise in antibiotic-resistant microorganisms and the emergence prevalence of immunocompromised diseases have contributed to an increasing number of refractory cases that challenge standard treatments [2,3,4].

Currently the most used classification is the Zurich Classification System (1991). It distinguishes three major types based on clinical appearance, disease duration, and radiological features:

1. Acute Osteomyelitis (AO)—duration less than 4 weeks.
2. Secondary Chronic Osteomyelitis (SCO)—duration of more than 4 weeks, often evolving from an acute episode, marked by milder symptoms.
3. Primary Chronic Osteomyelitis (PCO)—a non-suppurative, idiopathic chronic inflammation, typically presenting with intermittent flare-ups intersected with symptom-free periods.

Further classification may involve histological confirmation, particularly in atypical cases, and etiologic/pathogenic factors, which help guide treatment strategies.

Initial imaging with orthopantomography is recommended to evaluate dentition, detect signs of osteomyelitis, and rule out other pathologies or predisposing factors like fractures or bone diseases. When surgical intervention is considered, high-resolution computed tomography (CT) and/or dental scans are necessary to determine the degree of cortical bone destruction, identify sequestrum, and plan the extent of surgical resection. CT imaging is the examination of choice for distinguishing the more diffuse pattern of primary chronic osteomyelitis from the localized nature of secondary chronic osteomyelitis.

Management of jaw osteomyelitis is based on eradicating infection, controlling pain, preventing disease progression, and preserving anatomic structures. Empiric antibiotic therapy should be initiated and later adjusted based on culture and sensitivity results. Surgical intervention includes removal of infection sources such as infected teeth, osteosynthesis material or implants, and sequestrectomy. Depending on disease severity—assessed through clinical presentation and imaging findings—more extensive resection may be warranted. The extent of surgical resection and the method of reconstruction should be determined based on the individual case.

In primary osteomyelitis, despite the involvement of the bone, extensive resection does not guarantee effective disease control [2,3,4,5,6,7,8].

### Objectives

The aim of this study is to analyze a 14-year period of cases of osteomyelitis of the jaws, treated in a tertiary unit of maxillofacial surgery, and to determine treatment strategy for osteomyelitis.

## 2. Materials and Methods

### 2.1. Study Setting

We conducted a retrospective cohort study in 53 patients with chronic osteomyelitis of the jaws in a Portuguese tertiary unit of maxillofacial surgery from January 2010 to December 2023.

### 2.2. Participants

The inclusion criteria included a duration longer than 4 weeks, histopathological diagnosis, and the presence of follow-up appointments. The pathology was reviewed by a pathologist to confirm the diagnosis of osteomyelitis with these features: intertrabecular edema, medullary fibrosis, reactive bone, chronic inflammation, and the presence of bacteria inside marrow spaces.

Exclusion criteria were acute cases and no histological confirmation.

Demographic and clinical characteristics, treatment, and progression of the disease were collected for each patient.

Osteomyelitis cases were classified according to the Zurich Classification System: secondary chronic osteomyelitis and primary chronic osteomyelitis.

### 2.3. Data Collection

The primary outcome measures included the type and duration (median) of antibiotic therapy, surgical intervention following biopsy, type of reconstruction when applicable, and treatment outcomes (clinical remission) for each etiology group. Clinical remission was defined as the absence of clinical symptoms.

The secondary outcome measures included demographic data, presence of immunocompromised disease, localization, etiology, clinical symptoms, microbiological test results, type of osteomyelitis (according to the *Zurich* classification), and follow-up duration.

### 2.4. Data Analysis

Statistical analysis was performed using SPSS version 27.0 (IBM). Patient characteristics were summarized with means ± SD for normally distributed variables, median with interquartile range (IQR) for non-normally distributed variables, and frequencies (%) for categorical variables.

## 3. Results

### 3.1. Demographic and Clinical

Fifty-three patients were included in this study over a period of 14 years.

Twenty-eight (52.8%) were female, 25 (47.2%) were male. The mean age at diagnosis was 55 years (5–90). Twelve patients (22.6%) were smokers and 4 (7.5%) had excessive alcohol consumption (Table 1).

About twenty-two (41.5%) had immunocompromised conditions: 8 were iatrogenic, 8 had cancer, 1 presented HIV, 1 was diabetic, 1 had rheumatoid arthritis, 1 had tuberculosis, 1 had hepatic disease, and 1 other had renal disease.

Forty-five osteomyelitis cases (84.9%) affected the mandible, 8 cases (15,1%) the maxilla, and 1 affected both jaws. Forty-seven (88.7%) presented secondary chronic osteomyelitis and 6 (11.3%) primary chronic osteomyelitis.

Among the 47 secondary chronic osteomyelitis cases, 18 (38.3%) were caused by medication-related osteonecrosis of the jaw, 7 (14.8%) were caused by osteoradionecrosis, 8 (17%) were due to bone disease, 8 (17%) had an alveolar–dental etiology, 3 (6.3%) due to trauma (2 without osteosynthesis and 1 with osteosynthesis), 1 (2.1%) was due to periimplantitis, 1 (2.1%) was due to bone graft (2.1%), and 1 (2.1%) was post-orthognathic surgery.

At the first presentation of the disease, 49 (92.5%) reported pain, 43 (81.1%) had swelling, 15 (34.9%) had limited mouth opening,9 (17%) had hypoesthesia, 23 (43.4%) had intra-oral and/or extra-oral fistula, and 35 (66%) had bone exposure.

### 3.2. Management

#### 3.2.1. Diagnosis

Forty-six patients did a CT scan, only 3 did an orthopantomography and 4were without imaging (Table 2).

Bacteriological samples were taken in 25 cases (47.2%) and were contributory in 13 cases (52%). The isolated pathogens were *Actinomyces* in 6 patients (46.2%) and *Streptococcus* in 5 patients (38.5%). The other isolated germs are described in Table 1. From the isolated germens cases (*n* = 13), 6 (46.1%) were resistant to amoxicillin.

#### 3.2.2. Treatment

All patients received antibiotic therapy. The most administered antibiotics were amoxicillin plus clavulanic acid in 31 cases (58.5%) and amoxicillin plus clavulanic acid plus metronidazole in 15 cases (28.3%). The median time of antibiotic therapy was 27.3 days (95% CI 7–240 days).

Forty (75.5%) patients were submitted to sequestrectomy, 5 (9.4%) to marginal mandibulectomy, and 8 (15.1%) to segmental mandibulectomy. Fifteen needed reconstruction: 9 (60%) with a reconstruction plate, 2 (13.3%) with a bone graft, and 4 (26.7%) with a bone-free flap.

Seventeen (32.1%) had more than 2 surgeries.

Clinical remission was achieved in 41 (77.4%) patients over a median follow-up of 30.1 months (3–144 months). Among those who underwent sequestrectomy (*n* = 40), 31 patients (77.5%) had clinical remission. In the marginal mandibulectomy group (*n* = 5), 4 (80%) had clinical remission. In the segmental mandibulectomy group (*n* = 8), 6 (75%) achieved clinical remission.

In the primary chronic osteomyelitis group (*n* = 6), all individuals underwent sequestrectomy. Clinical remission was achieved in 5 cases (83.3%), with only 1 patient continuing to experience symptoms.

**Figure 1:**
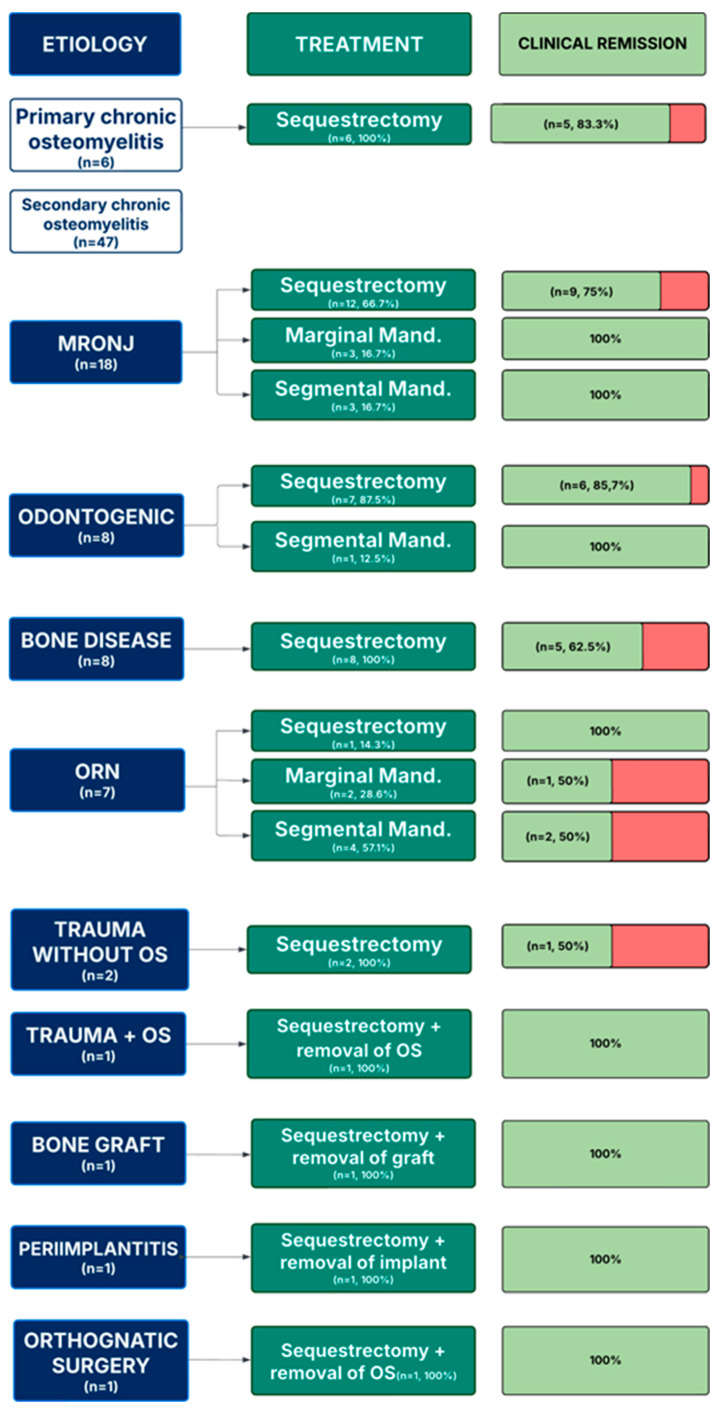
Treatment outcomes in patients with osteomyelitis per etiology.

In the medication-related osteonecrosis of the jaw (MRONJ) group (*n* = 18), 12 (66.7%) patients underwent sequestrectomy, while 3 (16.7%) required marginal mandibulectomy and another 3 (16.7%) underwent segmental mandibulectomy. All mandibulectomy cases were reconstructed using reconstructive plates. Clinical remission was achieved in 15 patients, whereas the remaining 3—who had only received sequestrectomy—continued to exhibit symptoms (Figure 1).

Among the odontogenic group (*n* = 8), sequestrectomy was performed in 7 (87.5%) patients, and 1 (12.5%) underwent segmental mandibulectomy. Clinical remission was observed in all but one case.

All patients in the bone disease group (*n* = 8) received sequestrectomy. Five (62.5%) reported clinical improvement, with (37,5%) maintaining unresolved symptoms.

Within the osteoradionecrosis (ORN) group (*n* = 7), surgical management included sequestrectomy in 1 case (14.3%), marginal mandibulectomy in 2 (28.6%), and segmental mandibulectomy in 4 (57.1%). Reconstruction involved fibula free flap in 4 cases and reconstructive plates in 2. Four individuals showed clinical remission, while 3 continued to experience persistent symptoms postoperatively.

For trauma without osteosynthesis (*n* = 2), sequestrectomy was the sole intervention. In one case the surgery did not show clinical improvement.

Cases associated with trauma and osteosynthesis, as well as post-orthognathic surgery, were addressed by sequestrectomy along with hardware removal with clinical remission.

In bone graft-related osteomyelitis case, sequestrectomy with removal of the graft showed clinical improvement.

The peri-implantitis case was managed through implant removal combined with sequestrectomy, with clinical remission.

## 4. Discussion and Conclusions

In our retrospective analysis of 53 chronic osteomyelitis cases, there was a slight female predominance (52.8%), consistent with findings by Daramola, Ajagbe, and Khullar et al., though contrasting with other studies reporting male predominance [2,3,5,6,7,8]. The mean age of onset was 55 years, slightly higher than previously reported averages [2].

The mandible was the most frequently affected site, consistent with the literature, due to its compromised vascularity, the cortico-spongy structure, and low mucosal coverage. [2,3,5,6,7,8] MRONJ was the most frequent cause of jaw osteomyelitis, followed by odontogenic infections, bone diseases, and osteoradionecrosis—differing from other reports where odontogenic infections are most common. This may reflect our histopathological inclusion criteria and the increased bisphosphonate use [9,10,11,12,13,14].

Almost half of the patients were immunocompromised, underscoring its role in osteomyelitis susceptibility [11,14]. Pain and swelling were the most common symptoms (>80%), while bone exposure (66%) and fistula formation (43.3%) suggested delayed diagnosis [2].

Bacteriological samples were collected in 25 cases (47.2%), with isolation of pathogens in 13 (52%) of these. The identified organisms were predominantly oral cavity flora, consistent with previously reported findings in the literature [2]. Non-contributory samples (*n* = 4) were a consequence of the difficulty collecting exploitable bacteriological oral specimens and avoiding contamination.

Almost half (46.1%) of the isolated germens cases demonstrated resistance to amoxicillin, reinforcing the need for routine antimicrobial susceptibility testing in guiding effective therapy. In the current context of rising antimicrobial resistance, the role of surgical intervention—particularly through debridement—becomes increasingly crucial in treatment planning. These findings raise important considerations regarding whether broader-spectrum or combination antibiotic therapies should be implemented to better address potential resistance patterns [15,16,17,18].

While the duration of antibiotic therapy of treatment varies according to the underlying etiology, it typically exceeds 3 weeks. In our cohort, most patients were treated with amoxicillin–clavulanic acid with a median antibiotic duration of 27 days. Notably, a shorter treatment period has also been explored; for instance, Lim R. found no statistically significant difference in outcomes between treatment durations less than 6 weeks and those longer than 6 weeks [18].

Osteomyelitis refractory to antibiotic therapy is guided by the etiology and severity of disease.

**Figure 2:**
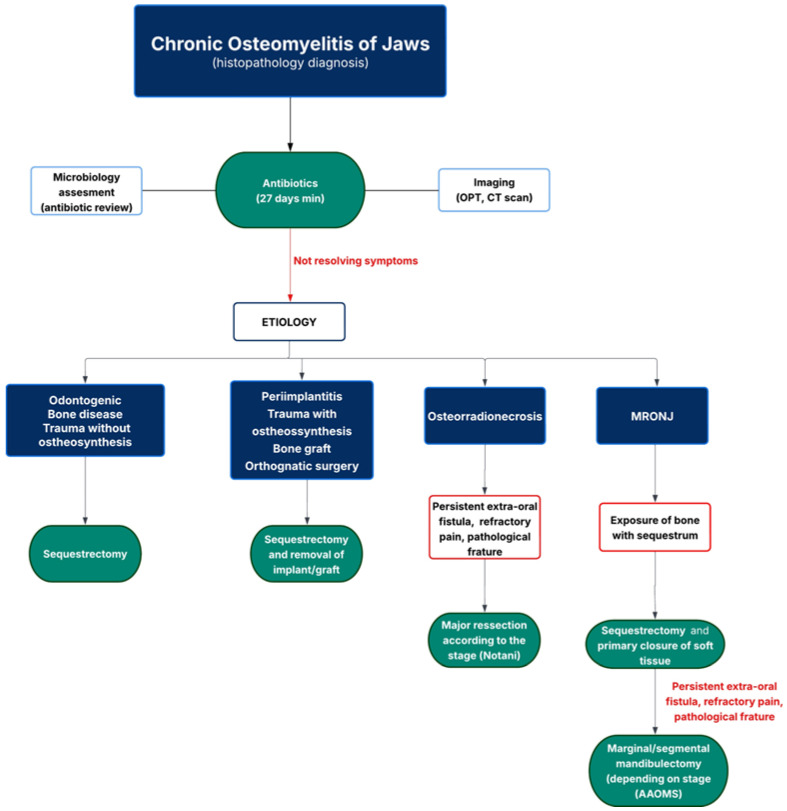
Flowchart of treatment management in chronic osteomyelitis of jaws.

In the MRONJ group, the predominant surgical approach was sequestrectomy (*n* = 12). However, in cases with more extensive bone involvement, marginal (*n* = 3) and segmental mandibulectomies (*n* = 3) were indicated. All mandibulectomy procedures were followed by reconstruction with reconstructive plates. Clinical remission was achieved in most patients (*n* = 15), whereas persistent symptoms were noted exclusively in 3 patients submitted to sequestrectomy. These findings suggest that while conservative debridement may be effective in select cases, more extensive surgical resection may be required for complete resolution in advanced MRONJ with osteomyelitis (Figure 2). It is also important to have full coverage of the bone with primary closure of the soft tissue, considering the pathogenesis of the disease [9,10].

In the ORN group, surgical management was generally more aggressive, consistent with the known pathophysiological complexity and impaired healing associated with irradiated bone. Segmental mandibulectomy was performed in 4 cases, while 2 patients underwent marginal mandibulectomy. Reconstructive strategies included fibula free flap transfer in 4 patients and reconstruction plates in 2, underscoring the necessity for vascularized bone in severe ORN. Despite the extent of surgical intervention, 3 patients continued to report symptoms postoperatively, highlighting the therapeutic challenges posed by ORN and the potential for persistent morbidity despite aggressive management. This contrasts with findings reported by *Marschall JS*, who achieved favorable outcomes using segmental mandibulectomy with immediate reconstruction. However, it is important to consider the underlying etiology of osteomyelitis, as the clinical manifestations of ORN are often complex and difficult to manage effectively [19,20].

MRONJ, ORN, and osteomyelitis have distinct pathophysiology but often present similar clinical, radiographic, and histopathological features. Superimposed infection and development of osteomyelitis are common in both MRONJ and ORN. Surgical debridement of devitalized tissue is recommended to enhance antibiotic penetration and provides an opportunity to collect bone samples for accurate histopathological and microbiological diagnosis [19,20].

Patients with osteomyelitis secondary to trauma with osteosynthesis, post-orthognathic procedures, and peri-implantitis were managed effectively through removal of necrotic bone in addition to hardware or implant extraction.

In cases of primary chronic osteomyelitis, all patients underwent sequestrectomy, with symptomatic resolution achieved in 83.3%. The favorable response to conservative surgical debridement in this subgroup aligns with current evidence, indicating that primary chronic osteomyelitis may be effectively controlled through targeted removal of infected bone in the absence of systemic comorbidities or complex structural involvement [21].

To optimize therapeutic outcomes and maintain clinical remission, we recommend a treatment management protocol outlined in the flowchart diagram demonstrated in Figure 2.

A major strength of this study is the strict inclusion criteria requiring histopathological confirmation of osteomyelitis, which enhances diagnostic accuracy and reduces the risk of misclassification [20]. With 53 patients included, this represents one of the larger single-center studies focused exclusively on chronic osteomyelitis of the jaw, enhancing the reliability of descriptive statistics. This study reflects a recent cohort, making its findings particularly relevant to current surgical management practices, especially considering the increasing prevalence of MRONJ.

The limited number of patients and the retrospective nature of our report constitute serious biases. The findings of this study have potential implications for clinical practice, particularly in the diagnostic and therapeutic approach to chronic osteomyelitis of the jaw.

The data support a tailored surgical approach guided by disease severity and underlying cause, reinforcing the role of more aggressive treatment in ORN cases.

Future research should prioritize prospective, multicenter studies with larger cohorts to improve data quality. The development of standardized diagnostic criteria combining clinical, radiographic, microbiological, and histopathological features would aid early and accurate diagnosis, especially distinguishing osteomyelitis features in MRONJ and ORN. Further research is needed to establish surgical guidelines, particularly regarding when to escalate from conservative debridement to segmental resection.

## Figures and Tables

**Table 1:** Demographic and clinical characteristics of the osteomyelitis population. Data are presented as *n* (%) unless stated otherwise.

	Total *n* = 53
**Age at diagnosis, y**	
Median (IQR)	55 (5–90)
**Sex**	
Male	25 (47.2)
Female	28 (52.8)
**Smoking Habits**	
Yes	12 (22.6)
No	41 (77.4)
**Alcohol habits**	
Yes	4 (7.5)
No	49 (92.5)
**Jaw affected**	
Mandible	45 (84.9)
Maxilla	8 (15.1)
Both	1 (1.9)
**Immunocompromised condition**	22 (41.5)
Iatrogenic	8 (36.3)
Cancer	8 (36.3)
HIV	1 (4.5)
Diabetes	1 (4.5)
Rheumatoid Arthritis	1 (4.5)
Tuberculosis	1 (4.5)
Hepatic disease	1 (4.5)
Renal disease	1 (4.5)
**Etiology**	
**Primary chronic osteomyelitis**	6 (11.3)
**Secondary chronic osteomyelitis**	47 (88.7)
Medication related osteonecrosis of the jaw	18 (38.3)
Osteoradionecrosis	7 (14.8)
Bone disease	8 (17)
Trauma	3 (6.3)
Alveolar–dental	8 (17)
Periimplantitis	1 (2.1)
Bone graft	1 (2.1)
Post orthognathic	1 (2.1)

**Table 2:** Diagnosis imaging and bacteriological sample. Data are presented as *n* (%) unless stated otherwise.

	Total *n* = 53
**Imaging**	49 (92.5)
CT scan	46 (93.9)
OPG	3 (6.1)
**Bacteriological samples**	
Performed	25 (47.2)
Polymicrobial	4 (16)
Germens isolated	13 (52)
*Actinomyces*	6 (46.2)
*Streptococcus*	5 (38.5)
*Klebsiella pneumoniae*	2 (15.4)
*Staphylococcus*	1 (7.7)
*Citrobacter braakii*	1 (7.7)
*Hafnia alvei*	1 (7.7)
*E* *ikenella corrodens*	1 (7.7)
*Proteus mirabilis*	1 (7.7)
*Prevotella buccae*	1 (7.7)
*Morganella morganii*	1 (7.7)

## Data Availability

The original contributions presented in this study are included in the article. Further inquiries can be directed to the corresponding author.
